# Associations Between Childhood Abuse and COVID-19 Hyperarousal in Adulthood: The Role of Social Environment

**DOI:** 10.3389/fpsyg.2021.565610

**Published:** 2021-02-24

**Authors:** Neha A. John-Henderson, Cory J. Counts, Annie T. Ginty

**Affiliations:** ^1^Department of Psychology, College of Letters and Science, Montana State University, Bozeman, MT, United States; ^2^Department of Psychology and Neuroscience, College of Arts & Sciences, Baylor University, Waco, TX, United States

**Keywords:** childhood abuse, hyperarousal, social support, COVID-19, PTSD, adults

## Abstract

**Background:**

Childhood abuse increases risk for high levels of distress in response to future stressors. Interpersonal social support is protective for health, particularly during stress, and may be particularly beneficial for individuals who experienced childhood abuse.

**Objective:**

Investigate whether childhood abuse predicts levels of posttraumatic stress disorder (PTSD) symptoms related to the COVID-19 pandemic, and test whether the perceived availability of social companionship preceding the pandemic moderates this relationship.

**Methods:**

During Phase 1, adults (*N* = 120; Age *M*[SD] = 19.4 [0.94]) completed a retrospective measure of childhood adversity along with a measure of perceived availability of opportunities for social engagement immediately preceding the pandemic. Two weeks after the COVID-19 pandemic declaration, participants completed the Impact of Event Scale-Revised (IES-R) with respect to the pandemic. Hierarchical linear regression analyses examined the interaction between childhood abuse and the perceived availability of social companionship preceding the pandemic as a predictor of PTSD symptoms.

**Results:**

Adjusting for covariates, the interaction between childhood abuse and perceived availability of others to engage with before the onset of the pandemic was a significant predictor of IES-hyperarousal (β = −0.19, *t* = −2.06, *p* = 0.04, Δ*R*^2^ = 0.032, CI: [−0.31 to −0.01]).

**Conclusion:**

Levels of perceived opportunities for social companionship before the pandemic associates with levels of hyperarousal related to the pandemic, particularly for individuals who experienced high levels of childhood abuse. More research is needed to understand how to mitigate the higher levels of distress related to the pandemic for these individuals in order to reduce risk for future psychiatric disorders.

## Introduction

A robust body of work highlights the long lasting implications of childhood abuse for health-relevant behaviors and outcomes into adulthood (e.g., Springer et al., 2003; Maniglio, 2009; De Bellis and Zisk, 2014; Beilharz et al., 2020). For example, trauma and abuse during childhood are risk factors for psychological distress and psychiatric disorders in adulthood (Duncan et al., 1996; Yehuda et al., 2001; Min et al., 2007; Shonkoff et al., 2009; Rogosch et al., 2011; Edwards et al., 2014; Nemeroff, 2016). It is theorized that in response to early life stress, the brain coordinates and regulates behaviors and physiological responses to stress in order to help the individual adapt to the demands of their environment (Hostinar and Gunnar, 2013). The experience of abuse is embedded into the regulation of stress systems in a way that shapes responses to future stressors (Berens et al., 2017).

While it is well known that a single stressor or stressful event can produce an ongoing cascade of event-related pathology, stressful events impact individuals differently (Lewis, 1992; Meaney et al., 1993; Bowman, 1997; Kirschbaum et al., 2008). For some individuals, their response to the stressor could result in a diagnosis of posttraumatic stress disorder (PTSD) or subclinical symptomology. Previous work indicates that childhood abuse is one factor which sensitizes individuals to future stressful events (Harkness et al., 2006; McLaughlin et al., 2010; Gouin et al., 2012; Nakai et al., 2014; Shapero et al., 2014; Asselmann et al., 2018). For example, in children who have experienced a natural disaster, previous exposure to trauma is an important predictor of post-disaster traumatic stress (Neuner et al., 2006). Separately, in veterans, reports of childhood abuse were higher in veterans with combat related PTSD compared to rates of PTSD in veterans who did not have combat-related PTSD, a relationship that was independent of levels of combat exposure (Bremner et al., 1993). Furthermore, a separate investigation found that childhood abuse changes psychological responses to future trauma, with greater reports of childhood abuse associating with higher levels of shame following the experience of a violent crime (Andrews et al., 2000). Together these findings provide compelling evidence that the experience of childhood abuse shapes the way in which individuals respond to future traumas or stressors.

A separate body of research links perceptions of social support to a variety of health outcomes (Cohen and Syme, 1985; Theorell et al., 1995; Brady and Helgeson, 1999; Wang et al., 2003; Uchino, 2006, 2009; Reblin and Uchino, 2008; Taylor, 2011). A perceived lack of social support and companionship can exacerbate illness and has been identified as a risk factor for poor psychological well-being (Avison and Gotlib, 1994; Cohen et al., 1997). According to the stress buffering hypothesis (Cohen and Wills, 1985), interpersonal social support can have a positive impact on health through preventing or dampening stress responses. For example, following a natural disaster, perceived social support was found to moderate the stressor–distress relationship, with individuals who reported lower levels of social support reporting more disaster related distress (Arnberg et al., 2012). Separately, previous findings indicate that social support and resources may be particularly important in the context of stress or adversity (John-Henderson et al., 2015), and in a prospective investigation, social support was found to mediate and moderate long-term consequences of childhood maltreatment (Sperry and Widom, 2013). Levels of interpersonal support and social engagement prior to a stressful event, may be particularly important in shaping psychopathology during uncertain times, and may be most important for individuals who have experienced early life trauma.

Based on previous work, infectious disease outbreaks elicit psychological responses (Sim et al., 2010; Shah et al., 2020; Xiang et al., 2020). The uncertainty which characterizes these outbreaks can contribute to increased levels of distress and PTSD symptoms (Cheng and Cheung, 2005; Lee et al., 2018). However, similar to other stressors, the nature of these psychological responses can vary significantly across individuals (Lau et al., 2007; Williams et al., 2012). On March 11, 2020, the novel coronavirus disease (COVID-19) was labeled as a pandemic and was acknowledged as a national emergency in the United States 6 days later. In an effort to reduce the spread of infection, recommendations were issued to stay home and socially distance, prompting individuals to make drastic changes to their daily lives and social environments. In general, previous work indicates that social companionship or the perceived availability of others to socially engage with is a predictor of physical and mental health (Seeman, 1996; Hale et al., 2005; Ang, 2018). However, it is unknown whether these perceptions would affect levels of distress in the context of a pandemic.

Merging these bodies of work, we hypothesize that childhood abuse may predict greater distress symptoms at the onset of the COVID-19 pandemic, in particular for those individuals who reported low levels of interpersonal support preceding the pandemic. While there are many dimensions of social support, we chose to focus on interpersonal support as reflected by the perceived availability of persons with whom one could engage in activities, because this social resource may be particularly protective as individuals enter a period of time where opportunities for social engagement are limited. In the context of the COVID-19 pandemic, it is possible perceived social companionship before social distancing recommendations were implemented, shaped the degree to which the stressor and associated changes in the social environment affected subsequent mental health.

## Methods

Participants (*N* = 120, 72% female; mean [standard deviation] age = 19.4 [0.94] years, age range 18–24 years; 69.3% White) were a subsample of an ongoing research study (total participants contacted from ongoing study *N* = 457). Exclusion criteria of the original study included a history of cardiovascular disease or current illness/infection. The initial laboratory visit (Phase 1) took place in Central Texas between January 2019 and February 2020. During this phase demographics, reports of childhood abuse, and perceived availability of social engagement and companionship were measured. During the second part of the study (Phase 2) participants resided in 22 different states during the follow-up. Participants completed questionnaires regarding pandemic related distress between March 26, 2020 and April 5, 2020. None of the participants had tested positive for COVID-19 and 87.5% of participants were living in a city/state that had a “shelter in place” order at the time of phase 2 completion. One participant was excluded due to incomplete data. All participants gave informed consent for both phases of the study. For Phase 1, participants were recruited through the university’s SONA system and received course credit. For Phase 2, all participants who completed Phase 1 were contacted and asked to take part in this second study. Participants who chose to take part were entered in a raffle to win one of 15, $25 gift cards. The studies were approved by the university’s Institutional Review Board.

### Questionnaires Phase 1

Childhood abuse was measured with the 28-item version of the Childhood Trauma Questionnaire (CTQ; Bernstein and Fink, 1998). The CTQ short form is a self-report measure which encompasses experiences from 0 to 17 years of age. The CTQ has five subscales which measure emotional abuse, emotional neglect, physical abuse, physical neglect, and sexual abuse. Each item uses a five-point frequency of occurrence scale: (1) never true, (2) rarely true, (3) sometimes true, (4) often true, and (5) very often true. In previous work, the CTQ short form has been found to be reliable and valid (α = 0.61 - 0.95; Bernstein et al., 2003). Following previous work (McLaughlin et al., 2014) we calculated a threat score using subscales from the CTQ. Childhood threat has been associated with higher levels of future internalizing and externalizing symptoms (Miller et al., 2018) and high levels of psychological distress (Hughes et al., 2007; Llabre et al., 2015). The threat score consists of sum of the following subscales: physical abuse, sexual abuse, and emotional abuse. Cronbach’s alpha for the threat score in the present study was 0.617.

Interpersonal social support and resources were measured using the Interpersonal Support Evaluation List –Short version (ISEL-12; Cohen and Hoberman, 1983). The ISEL-12 is a self-report questionnaire with 12 statements regarding the perceived availability of social resources and support. We focused on the belonging subscale of the ISEL-12 which is meant to assess the perceived availability of persons to engage in social activities with (Cohen et al., 1985). An example item from the belonging scale is, “If I wanted to have lunch with someone, I could easily find someone to join me.” Participants respond to each question using the following scale: 1 (*definitely false*), 2 (*probably false*), 3 (*probably true*), and 4 (*definitely true*). Higher scores on this measure reflect a perceived greater ability to engage with others in social activity before the pandemic. The belonging subscale demonstrated good internal consistency in this sample (Cronbach αs > 0.790).

### Questionnaires Phase 2

Participants who had completed the larger study were sent an email on March 26, 2020 notifying them about the opportunity to participate in a follow-up study online. In this follow-up they completed the Impact of Events Scale-Revised (IES-R), which is used to assess subjective distress and PTSD symptomology for a specific event (Weiss and Marmar, 1997). In the present study, the life event was the COVID-19 coronavirus pandemic. The IES-R has three subscales, intrusion (e.g., intrusive cognitions, nightmares), avoidance (e.g., avoidance of feelings), and hyperarousal (e.g., anger, trouble concentrating). Item response anchors for this scale are: 0 (*not at all*), 1 (*a little bit*), 2 (*moderately*), 3 (*quite a bit*), and 4 (*extremely*). Each subscale score is computed as the mean item response of the subscale item. The IES-R subscales have good internal consistency in previous work (Craparo et al., 2013) and in this research (Cronbach αs > 0.791).

### Statistical Analysis

All statistical analyses were conducted using SPSS (version 24; IBM, Armonk, NY). We first examined descriptive statistics and bivariate Pearson correlations between the CTQ threat score, ISEL-belonging scores, IES-R subscales (intrusion, avoidance, and hyperarousal), and demographics. Next, in three separate hierarchical linear regressions, adjusting for age, race, biological sex, and depressive symptoms, we examined whether childhood abuse interacts with perceived availability of others for social engagement preceding the pandemic to predict clusters of PTSD symptoms related to the COVID-19 pandemic. To identify specific values of CTQ threat for which the relationship between ISEL-belonging and IES-hyperarousal is statistically significant in this sample, we used the Johnson-Neyman technique (Rast et al., 2014).

## Results

### Descriptive Statistics

One hundred and twenty participants completed both phases of the study. Participants had a mean (SD) HADS score of 4.25 (2.78) and a CTQ threat score of 19.70 (5.72) during phase 1 of the study. The mean (SD) parental occupational status was 5.94 (1.20). During phase 1 of the study, participants reported an ISEL belonging mean (SD) of 8.82 (2.51). During phase 2 of the study, participants reported mean (SD) scores for the following subscales of the IES-R: hyperarousal, 0.84 (0.74), intrusion 0.86 (0.73), and avoidance 1.14 (0.72). See Table 1 for mean, standard deviation, and ranges of demographic information and descriptive statistics.

**TABLE 1:** Descriptive statistics.

	**Mean (*N* = 120)**	**SD/%**	**Range**
Age	19.40	0.94	18.11–24.50
Sex		72.4% (Female)	–
Race		69.3% (White)	–
Parental occupation status	5.94	1.20	2–7
Current depressive symptoms	4.25	2.78	0–32
Childhood threat score	19.70	5.72	15–52
ISEL belonging	8.82	2.51	3–14
IES-R hyperarousal	0.84	0.74	0–3.17

*Parental occupation was coded as: 2 = unskilled, 3 = partly skilled, 4 = skilled manual, 5 = skilled non-manual, 6 = skilled managerial, and 7 = professional. ISEL: Interpersonal Support Evaluation List; IES-R: Impact of Events Scale-Revised.*

### Correlation Analyses

Higher levels of CTQ threat were moderately associated with lower socioeconomic status (SES) and higher depressive symptoms. Females had higher levels of ISEL-belonging. Additionally, there was a moderate association between higher levels of ISEL-belonging were associated with lower levels of depressive symptoms. There was a small association between higher IES-intrusion and IES-hyperarousal associated with being older, having more depressive symptoms at time 1. There was a small association between high CTQ threat was associated with higher scores on all three IES subscale. Lastly, there was a moderate to large association between all three subscales of the IES were related to one another with moderate to large effects. Table 2 reports the full correlation matrix.

**TABLE 2:** Bivariate correlations of interest variables.

**Variable**	**1**	**2**	**3**	**4**	**5**	**6**	**7**	**8**	**9**
1. Age	–								
2. Sex	−0.26**	–							
3. Depressive symptoms	0.19*	0.12	–						
4. SES	–0.05	–0.14	−0.20*	–					
5. ISEL-belonging	–0.07	0.23*	−0.42**	0.07	–				
6. CTQ threat	0.05	–0.01	0.21*	−0.30**	–0.16	–			
7. IES-intrusion	0.21*	0.12	0.21*	–0.01	–0.01	0.25**	−		
8. IES-avoidance	0.06	0.17	0.16	–0.08	–0.01	0.20*	0.64**	–	
9. IES-hyperarousal	0.19*	0.09	0.32**	–0.07	–0.07	0.25**	0.84**	0.62**	–

Sex: 1 = male, 2 = female; SES: Socioeconomic status; ISEL: Interpersonal support Evaluation List; IES: Impact of Events Scale.

*Correlation significant at the 0.05 level (two-tailed).

**Correlation significant at the 0.01 level (two tailed).

### Hierarchical Regressions

We conducted a series of hierarchical regression to test our main hypothesis. In block 1 we entered age (calculated from reported date of birth), race, biological sex, depressive symptoms, and parental occupation status along with the CTQ threat score and the ISEL-belonging subscale score. In block 2, we entered the interaction term for CTQ threat and ISEL-belonging. We examined whether this interaction term was a significant predictor of IES-intrusion, IES avoidance and IES hyperarousal. The interaction term was not a significant predictor of IES intrusion or IES avoidance (β = −0.064, *t* = −0.70, *p* = 0.48 and β = −0.042, *t* = −0.44, *p* = 0.66, respectively). However, we found that the interaction between CTQ threat and ISEL-belonging was a significant predictor of IES hyperarousal (β = −0.19, *t* = −2.06, *p* = 0.04, Δ*R*^2^ = 0.032, CI: [−0.31 to −0.01]). This regression model is reported in Table 3. Using the Johnson-Neyman technique (Rast et al., 2014), we found that the relationship between ISEL-belonging and IES-hyperarousal was statistically significant for participants who had CTQ threat scores above 28.63 (possible range: 15–75, observed range: 15–52). Specifically, for individuals with high levels of childhood abuse, low levels of ISEL-belonging predicted higher levels of IES-hyperarousal.

**TABLE 3:** Predictors of IES-R Hyperarousal in hierarchal linear regression model.

	**IES hyperarousal**		
	**β**	***p***	**Δ*R*^2^**
Age	0.15	0.11	
Sex	0.08	0.41	
Race	−0.16	0.07	
Parental occupation status	0.02	0.87	
Depressive symptoms	0.27	0.01	
Childhood threat score	0.21	0.02	
ISEL belonging	−0.07	0.48	
Childhood threat score × ISEL belonging	−0.19	0.04	0.032

*N = 120.*

## Discussion

The findings reported here utilize data which were collected before the onset of the COVID-19 pandemic and data from a follow-up which took place during the weeks immediately following its classification as a national emergency in the United States. In a sample of 119 adults, we found that for individuals who reported experiencing high levels of childhood abuse, there was a significant relationship between perceived availability of social companionship preceding the pandemic and distress symptoms related to the pandemic. Specifically, these individuals had higher levels of hyperarousal symptoms related to the pandemic. This cluster of symptoms is characterized by constantly feeling on guard, having difficulty falling or staying asleep, feeling jumpy or easily startled, and feeling irritable.

Given that individuals who experience childhood abuse are known to be at greater risk for psychiatric disorders in adulthood compared to individuals who did not experience childhood abuse (Duncan et al., 1996; Shonkoff et al., 2009; Rogosch et al., 2011; Nemeroff, 2016), it is important to understand the risk and protective factors which moderate the relationship between stressful events, PTSD symptoms and subsequent risk for PTSD for this population. Our findings suggest, in the context of the COVID-19 pandemic, high levels of perceived availability of social companionship preceding the pandemic is one such factor which may reduce subsequent risk. It is possible that efforts to increase opportunities for social engagement generally for this at-risk population may help to offset high levels of PTSD symptoms in response to stressful events. Further, during stressful events such as the ongoing COVID-19 pandemic, it is possible that efforts to increase social engagement, even if virtually due to social distancing recommendations, may help to manage their pandemic related distress.

The findings are in line with a body of work indicating a relationship between childhood trauma and risk for psychiatric disorders into adulthood (Widom, 1999; Copeland et al., 2007; Widom et al., 2007). Individuals who experience abuse during childhood exhibit chronically high levels of corticotropin releasing factor (CRF) as adults, which causes generalized arousal, anxiety and hypervigilance, all of which are symptoms of the PTSD hyperarousal cluster (Charney et al., 1993). While we do not measure CRF or other biological proteins which may contribute to observed hyperarousal symptoms, our findings indicate that the relationship between childhood maltreatment and this cluster of PTSD symptoms in response to stress may be moderated by one’s social environment preceding the stressor. In light of this observation, it may be important to consider social environments more closely when assessing risk for PTSD following a stressor for this at-risk population.

The findings from this research could have clinical implications for the development of interventions which could benefit adults who experienced childhood abuse, specifically in the context of a life event such as the COVID-19 pandemic. Based on the pattern of observed findings, interventions which provide these adults with opportunities for social engagement could be useful in dampening distress levels related to the event. In the context of live events which make in-person social engagement challenging (e.g., the COVID-19 pandemic), social engagement opportunities could be made available online or using social media platforms.

This research has important limitations. First, while the study is prospective, it is still correlational, and it remains possible that the pattern of our results are affected by a separate variable or construct (Christenfeld et al., 2004). However, the pattern of results we observed accounted for variables which may be related to our outcome variables including age, biological sex, ethnicity and depressive symptoms. Second, since the symptoms measured using the IES-R had not persisted over a period of a month, the participants in this sample do not meet diagnostic and statistical manual of mental disorders (DSM)-V criteria and the IES-R is not an official diagnostic criteria instrument for PTSD.

Overall, our findings provide initial evidence, that low levels of perceived availability of social companionship and opportunities for social engagement with others preceding a stressful event, may be a social risk factor for subsequent development of subclinical PTSD symptoms and potentially future PTSD diagnosis.

## Data Availability Statement

The raw data supporting the conclusions of this article will be made available by the authors, without undue reservation.

## Ethics Statement

The studies involving human participants were reviewed and approved by the Baylor University Institutional Review Board. The participants provided their written and informed consent to participate in the study.

## Author Contributions

AG designed the research study, collected the data, and participated in writing the manuscript. CC helped with the data analyses and preparation of findings for the manuscript. NJ-H analyzed the data and wrote the manuscript. All authors contributed to the article and approved the submitted version.

## Conflict of Interest

The authors declare that the research was conducted in the absence of any commercial or financial relationships that could be construed as a potential conflict of interest.
